# Flood in the South-West of Iran in 2019; Causes, Problems, Actions and Lesson Learned

**DOI:** 10.29252/beat-070219

**Published:** 2019-04

**Authors:** Mahmoudreza Peyravi, Ali Asghar Peyvandi, Ali Khodadadi, Milad Ahmadi Marzaleh

**Affiliations:** 1 *Department of Health in Disasters and Emergencies, Health Human Resources Research Center, School of Management and Medical Informatics, Shiraz University of Medical Sciences, Shiraz, Iran.*; 2 *Hearing Disorders Research Center, Shahid Beheshti University of Medical Sciences, Tehran, Iran.*; 3 *Department of Immunology, School of Medicine, Ahvaz Jundishapur University of Medical Sciences, Ahvaz, Iran; Khuzestan Red Crescent, Ahvaz, Iran.*; 4 *Ph.D. Candidate of Health in Disasters and Emergencies, Student Research Committee, Department of Health in Disasters and Emergencies, Health Human Resources Research Center, School of Management and Medical Informatics, Shiraz University of Medical Sciences, Shiraz, Iran*

**Keywords:** Flood, Climate change, Health, Lesson learned, Iran

**Dear Editor,**

Extreme climatic changes have increased the frequency and intensity of natural disasters all over the world imposing disastrous effects on human life and environment[1]. One of the dangerous disasters is flood. Flood may happen due to extreme rain or human-made actions in shores[2]. This report has investigated the feature and causes, actions taken, problems faced and lessons learned regarding flood management in Khuzestan province in Iran in January, 2019.

*Features and Causes*

Extreme rain in January in Lorestan, Ilam, Kermanshah and Khuzestan provinces; led to the overflow of the catchment areas of Dez, Karun, Karkheh, Maroon and Zohreh dams. According to the estimates, 12775 million cubic meters of water entered the catchment areas of aforementioned dams from the 1st till the 10th of January 2019. In the 8th of January 2019, a great flood occurred in most regions of Khuzestan province. This flood led to the occurrence of extreme torrent and vast inundation in different regions and villages of Ahvaz, Shush, Dasht Azadegan, Hoveyzeh, Dezful and Shushtar, as well as increasing the water level of Karun, Dez and Karkheh rivers. These areas are the raining system located in the northern and the southern parts of Khuzestan province. Owning to the emancipation of Dez river water, this flood over flew and caused many damages to agricultural lands and residential areas of the north of Khuzestan. Broken flood protectors and lack of attention to their repairing and reconstruction by Khuzestan water and power authority led to the occurrence of flood in various villages of the province, and caused damages to the agricultural lands. Overall, the main causes of this phenomenon included: 1. Long lasting droughts and sedimentation during previous 5 years, 2. Building construction in rural and agricultural lands, revegetation basins and other human actions along rivers, 3. Reduction of the capacity of Hourol Azim marshes of Bamdezh due to various other human interventions and excavation activities, and 4. Destruction of flood protectors for having more access to water during drought 

*Actions taken*

The actions taken for management for reducing the impact of the flood included performing initial evaluations by the Red Crescent; monitoring the forecasting reports of Aerology Organization and Khuzestan water and power authority, alerting the readiness Crisis Management Committee of Khuzestan province, reducing the amount of water behind Dez and Karkheh dams; reconstructing flood protectors by people and; organizations in charge; professionally preventing the leakage of flood protectors by water and power authorities; providing emergency relief by Red Crescent to 3455 families; establishing several emergency camps in Khuzestan province by Red Crescent; and establishing a field hospital and sending relief helicopters to the flooded regions; distributing items like assistance tents, blankets, carpets and covering nylons; distributing monthly food packages among people; and transforming vulnerable individuals and appropriate cooperation with people.

*Problems faced*

While a huge effort was made in helping people by the Red Crescent, police, the Crisis Management Organization and Ministry of Health and Medical Education, there was lack of a coherent unit commander for managing the flood, resulting in incomplete evacuation of some villages, and absence of students from schools. This inundation results in 8000 Billion Rials pecuniary loss to flooded regions.

*Lesson Learned*

This exceptional incidence has strengthened our expertise and the following lessons for managing of similar conditions in future were learned: 

Prevention of interference and possession of active and torrential beds of the rivers in the province.

1. Enabling and training people toward logical use of dams and rivers is helpful.

2. Using protocols and standards during disasters leads to the fair distribution of resources among people.

3. Creating a unit commander would be helpful.

4. Given the water outage in the first hours, it is a must to equip the health and treatment centers with safe water supply systems for establishing communication when a disaster occurs.

5. There is a need to integrate service presentation and management of volunteers who may be scattered all over the region aimlessly.

6. Having an information record system and documents of all the presented services would lead to an effective response toward disasters.

7. Spiritual and psychological support of the flooded people should be done by responsible organizations.

8. Cautions must to be declared strongly and obligatorily to people and responsible organizations.

## Conclusion

 Irregular interference and possession of active and torrential beds of the rivers in the province, such as developing agricultural, cultivating trees and illegal constructions despite precautions of Khuzestan water and power authority by native persons and governmental organizations, has decreased the capacity of river conduits and eventually has caused flood distribution and inundation of residential and agricultural lands. Due to the specific climatic conditions in Iran, and the annual occurrence of various floods in different regions, necessary precipitation and planning should be done before the occurrence of these disasters by the organizations in charge. Urban and rural planners should take actions toward identify the dangers sites of the region and design tolerant substructures of torrential and runoff gatherings. Moreover, it is recommended to hold training courses for local people to empower them to manage similar disastrous events. National and local politicians can increase the people’s tolerance toward natural disasters through improving their capacities and reducing their vulnerability.

## References

[1] Chau VN, Holland J, Cassells S, Tuohy M (2013). Using GIS to map impacts upon agriculture from extreme floods in Vietnam. Applied Geography.

[2] Agbola BS, Ajayi O, Taiwo OJ, Wahab BW (2012). The August 2011 flood in Ibadan, Nigeria: Anthropogenic causes and consequences. International Journal of Disaster Risk Science.

